# Can centre-based childcare buffer against the negative effects of family adversity on child socio-emotional wellbeing?

**DOI:** 10.1093/eurpub/ckab006

**Published:** 2021-02-07

**Authors:** Alison Parkes, Michael Green, Anna Pearce

**Affiliations:** MRC/CSO Social and Public Health Sciences Unit, University of Glasgow, Glasgow, UK

## Abstract

**Background:**

Different configurations of family adversity affect children’s socio-emotional development differently; however, we lack knowledge of moderators amenable to policy intervention. This study explored whether early childhood centre-based childcare moderated the impact of family adversity configurations on socio-emotional development.

**Methods:**

Data were from the Growing Up in Scotland first birth cohort, born 2004–05. Latent class analysis of 19 early childhood family adversity indicators identified four classes: ‘Low Risk’ (68%), ‘Poor Maternal Health’ (16.5%), ‘Economic Hardship’ (10.0%) and ‘Multiple Adversities’ (5.5%). Latent growth models of externalizing and internalizing symptom trajectories (age 46–152 months, *n* = 3561) by family adversity controlled for confounding. Moderation by centre-based childcare use was examined through stratification.

**Results:**

Compared to ‘Low Risk’, high-risk classes had more externalizing and internalizing symptoms and internalizing symptoms increased at a faster rate, with ‘Multiple Adversities’ faring worst. The effects of ‘Economic Hardship’ on change in externalizing symptoms over time varied by childcare (*P *=* *0.035): relative to the Low Risk group, symptoms increased (+0.04 points/year) among those not using childcare, and decreased (–0.09 points/year) among those who did. The effect of ‘Multiple Adversities’ on internalizing symptoms also varied *(P *=* *0.034): +0.12 without centre-based childcare; +0.33 with centre-based childcare (patterns were similar for externalizing symptoms but with wide confidence intervals). No moderation was found by ‘Poor Maternal Health’.

**Conclusions:**

Centre-based childcare may alleviate disadvantages in socio-emotional wellbeing for children experiencing mainly economic hardship, but may exacerbate them for those experiencing multiple adversities. A better understanding of how early years’ services can support families with complex needs is required.

## Introduction

Cumulative childhood adversity including economic stress, disruptive family processes and poor parental mental health is associated with behavioural and emotional difficulties for children and poorer mental health in adulthood.^1–3^ Yet despite the compelling evidence base in relation to cumulative adversity, the assumption that risk factors are additive and interchangeable^4^ limits our understanding of appropriate policy tools to support families experiencing different combinations of social adversity and reduce inequalities in socio-emotional outcomes. Different patterns of family adversity are known to have different implications for socio-emotional development,^5–8^ but existing work has not yet explored moderators: clarifying what works best, and for whom, has the potential to unlock more nuanced policies and interventions. The main aim of this study is to investigate centre-based childcare as a moderator of associations between different patterns of family adversity in early childhood and children’s later socio-emotional outcomes, using a nationally representative population sample.

Formal early childhood education and care offered by centre providers such as nurseries, playgroups or family centres may benefit children’s development.^9^^,^^10^ Although centre-based childcare contributes to a ‘levelling up’ of the academic skills of children from disadvantaged families,^10^ it is less clear whether disadvantaged children’s socio-emotional adjustment benefits to the same extent. Some studies find support for compensatory effects, with formal childcare reducing the effects of single dimensions of disadvantage (economic strain, harsh parenting or maternal depression) on children’s internalizing and externalizing symptoms.^11–13^ In contrast, others suggest lesser benefits or greater harms of centre-based care for children from lower SES families.^14^^,^^15^

Greater clarity is needed on differential benefits and harms of centre-based childcare, especially as family disadvantage is often complex and multidimensional. Traditional moderation analyses of the effects of childcare on socio-emotional outcomes have each focused on a single dimension of disadvantage.^11–15^ One study of Head Start programme effectiveness provides a notable exception, signalling the importance of different configurations of family risk within the disadvantaged target sample.^16^ Further research is, however, needed on whether universally available centre-based childcare provision shows comparable variation in effect across different configurations of family adversity within a general population. Added value would also come from examining whether differential childcare effects persist into middle childhood, especially given recent evidence for long-lasting effects of childcare on all children.^10^

Our study used latent class analysis (LCA) to classify families according to their pattern of adversity in a large and nationally representative cohort and a wide range of indicators theorised by ecological models^17^ as having proximal risks for child development: these relate to economic stress, disruptive family processes, poor parental health and household change. We explore whether centre-based childcare in early childhood moderated the impact of different patterns of family adversity on socio-emotional outcomes from age 4–12.

## Methods

### Study participants

Data were from the first birth cohort of the Growing Up in Scotland (GUS) study,^18^ a nationally representative cohort of families with children born in 2004–05. Families registered for universal Child Benefits were eligible to take part. Geographical clusters of families were created and selected at random from strata of local authorities and area deprivation. Interviews were carried out in the home, with parents, and at later ages, the cohort members and their teachers. At the most recent (age 12) survey, just over one in three were overweight, life satisfaction was generally very high, one in five had tried alcohol, but only one in 20 had tried cigarettes.^19^ Further details of the sampling framework are provided elsewhere.^20^ Data collection was subject to medical ethical review by the Scotland ‘A’ MREC committee. All participants provided written informed consent. Further consent and ethical approval were not required for the secondary analyses presented in this paper.

We determined common patterns of family adversity during early childhood from interviews at 10, 22 and 34 months (*n* = 3928) excluding families who had dropped out by the third interview (*n* = 1097), those with multiple births (*n* = 78) or those where the mother was not interviewed at each time point (*n* = 114). After applying survey weights, this ‘Early Childhood sample’ closely resembled the baseline sample (Supplementary table S1). To examine associations between family adversity, centre-based childcare and children’s outcomes, we excluded 367 families in the Early Childhood sample where there was no information from mothers on children’s externalizing and internalizing symptoms at any timepoint from interviews at 46, 58, 70, 94, 122 and 152 months: leaving a ‘Main Analysis sample’ of *n* = 3561 families. Compared to the Early Childhood Sample, there were slightly fewer mothers with low educational qualifications, lacking a co-resident partner at baseline or in the lowest household income quintile. To address this, survey weights were multiplied by inverse probability weights of inclusion in the Main Analysis sample, thereby rendering it closely similar to the Early Childhood sample (Supplementary table S1). A flow diagram, summarizing how the Main Analysis Sample was arrived at, is provided in Supplementary figure S2.

### Measures

#### Outcomes: child externalizing and internalizing symptoms

Parent information was collected at interviews between child ages 46 and 152 months, using the Strengths and Difficulties Questionnaire (SDQ),^21^^,^^22^ As recommended,^23^ an externalizing score combined five items from the conduct subscale (temper tantrums, obedience, fights with other children, lies/cheats, steals) and five items from the hyperactivity subscale (restless/overactive, fidgets, distracted, thinks before acting, sees tasks through). The internalizing score combined five items from both the peer relationship subscale (solitary, has one good friend, liked by other children, picked on/bullied, gets on better with adults) and the emotional subscale (headaches, worries, unhappy, nervous/clingy, scared) problems subscales. Each item was assigned a value of 0–2 according to the following responses: Not True, Somewhat True, Certainly True (with positive items reversed coded). When totalled, the externalizing and internalizing scores both ranged from 0 to 20, with higher scores indicating more symptoms. The main analyses focused on these continuous scores (mean scores are presented, for all ages, in table 1). In descriptive analyses, we also examined ‘severe’ internalizing and externalizing behaviours, using cut-offs of 9 (externalizing) and 7 (internalizing). These cut-points summed the validated cut-points for each of the relevant SDQ components for borderline-abnormal scores.^21^ Supplementary analyses used additional outcome information collected in separate questionnaires from teachers of GUS children (122 months) and the children themselves (122 and 152 months). Further detail on the teacher survey methodology is provided elsewhere^24^; for further details of the variables used, see Supplementary File.

**Table 1:** Externalizing and internalizing symptoms in the total sample and according to family adversity class

				Family adversity class							
		Total sample		Low Risk		Poor Maternal Health		Economic Hardship		Multiple Adversities	
		mean (95% CI)	% severe	Mean (95% CI)	% severe	Mean (95% CI)	% severe	Mean (95% CI)	% severe	Mean (95% CI)	% severe
Externalizing symptoms											
Child age (months)	46	5.60 (5.48–5.73)	18.4	4.95 (4.83–5.07)	11.4	6.36 (6.14–6.59)	25.9	6.78 (6.35–7.21)	30.1	7.81 (7.13–8.49)	44.6
	58	5.58 (5.35–5.61)	17.7	4.75 (4.62–4.88)	9.7	6.36 (6.08–6.64)	28.4	6.59 (6.13–7.04)	27.8	8.00 (7.32–8.68)	46.9
	70	5.17 (5.04–5.31)	15.6	4.44 (4.33–4.56)	9.6	5.94 (5.64–6.24)	22.0	6.46 (5.90–7.03)	23.5	7.82 (7.13–8.51)	41.5
	94	5.04 (4.90–5.20)	16.6	4.30 (4.14–4.46)	10.8	5.90 (5.52–6.29)	22.9	6.27 (5.76–6.78)	24.9	7.78 (7.04–8.52)	40.3
	122	4.40 (4.23–4.58)	12.4	3.69 (3.54–3.83)	6.9	5.07 (4.71–5.44)	16.6	5.50 (4.79–6.21)	19.8	7.38 (6.54–8.22)	39.5
	152	4.38 (4.19–4.57)	12.9	3.67 (3.49–3.84)	7.6	5.16 (4.77–5.56)	17.1	5.62 (4.85–6.39)	19.8	7.27 (6.26–8.28)	43.8
Internalizing symptoms											
Child age (months)	46	2.22 (2.15–2.29)	5.6	1.95 (1.87–2.04)	3.2	2.95 (2.72–3.18)	9.3	2.73 (2.43–3.03)	8.3	3.58 (3.13–4.03)	15.2
	58	2.32 (2.22–2.43)	6.3	1.88 (1.80–1.95)	3.4	2.91 (2.67–3.15)	9.5	2.72 (2.42–3.02)	8.1	4.14 (3.56–4.73)	21.8
	70	2.24 (2.13–2.36)	6.7	1.81 (1.73–1.90)	4.1	2.76 (2.52–2.99)	9.6	2.74 (2.40–3.08)	7.9	4.17 (3.63–4.71)	21.3
	94	2.74 (2.58–2.90)	11.0	2.13 (2.01–2.25)	5.6	3.53 (3.18–3.87)	17.2	3.49 (2.96–4.02)	16.8	5.26 (4.35–6.18)	36.0
	122	3.00 (2.79–3.21)	13.9	2.37 (2.23–2.52)	8.6	3.80 (3.39–4.21)	19.7	3.74 (3.04–4.45)	21.3	5.56 (4.54–6.58)	35.8
	152	3.53 (3.31–3.76)	18.6	2.77 (2.59–2.95)	12.2	4.60 (4.04–5.15)	26.5	4.48 (3.63–5.32)	26.5	6.82 (5.64–7.99)	48.2

Notes: This table shows unadjusted associations between family adversity class and mother-reported symptoms at each measurement age. ‘Severe’ problems were defined using a cut-off of 9 for externalizing and 7 for internalizing.^21^

#### Main exposure: family adversity class

This was based on 19 binary indicators summarizing adversities experienced at child ages 10, 22 and 34 months, including: four indicators of persistent economic hardship (income < 60% of median, in receipt of income support, workless household, financial stress); four indicators of disruptive family processes (partner conflict, partner separation, new partner, harsh parenting); six indicators of maternal health problems (low mental health, depression and stress, frequent alcohol use, illegal drug use, low physical health, limiting illness/disability) and five indicators of major household events: family illness/accident, new child in household, child left household, death of grandparent/other close relative, moved house. Further details are provided in Supplementary table S2. LCA using Mplus version 8^25^ identified family adversity classes, allowing for the complex survey design. Various model fit statistics were used to help identify the optimum number of classes, together with considerations of whether classes were distinctive in nature, and large enough to investigate further.^26^ A four-class solution was identified as the best fit on the basis of the Lo, Mendell and Rubin Likelihood Ratio test (for model fit statistics, see Supplementary table S3). Classes were labelled according to their main source(s) of adversity as: Low Risk (68%), Poor Maternal Health (16.5%), Economic Hardship (10.0%) and Multiple Adversities (5.5%). Supplementary figure S1 in the supplement shows indicator distributions across each class assigned on the basis of highest class probability. Cumulative risk scores derived by summing indicator probabilities were Low Risk 1.5, Poor Maternal Health 3.8, Economic Hardship 5.0 and Multiple Adversities 7.9.

A five-class solution was also explored: this subdivided maternal health problems into mental and physical health problems. As findings for these classes were closely similar, we opted for the more parsimonious four-class model.

#### Potential moderator: centre-based childcare

At 10, 22 and 34 months, mothers supplied information on all regular childcare arrangements for their child. Nurseries, crèches, playgroups and family centres were classed as ‘centre’ providers, while individuals such as grandparents, friends, childminders and nannies were classed as ‘non-centre’. A binary measure of exposure to centre-based childcare was based on any use at 10, 22 and/or 34 months (yes/no). Around half of families in the Main Analysis sample used centre-based childcare: median usage across all three time points was 12 hours per week. Use was lower among families in the Economic Hardship (40%) and Multiple Adversities classes (42%) compared to those in the Poor Maternal Health (52%) and Low Risk (53%) classes.

#### Covariates

The following were selected as baseline demographic confounders of associations between family adversity class, centre-based childcare and children’s behavioural and emotional symptoms: maternal education (low: lower level Scottish Standard grades, or none; intermediate: upper level Standard grades/Scottish Highers; high: degree or equivalent), maternal ethnicity (white/minority, due to low numbers of ethnic minority groups), maternal age at birth of cohort child (under 20, 20–29 years, 30+ years), maternal smoking in pregnancy (yes/no), mother is single parent (yes/no), family size (1, 2, 3+children), child sex, low birthweight, time spent in neonatal unit. Non-centre-based childcare (hours per week averaged across 10, 22 and 34 months) was also included as a potential confounder.

### Analysis

Associations between family adversity class membership and children’s externalizing and internalizing symptom trajectories were explored, using parallel process latent growth curve modelling in Mplus version 8.^25^ We model a linear slope of parent-reported symptoms from 46 to 152 months, and a quadratic slope, to allow curvature in the relationship (or in other words, for an upward or downward change to the linear slope with age). The intercept was initially set at the trajectory midpoint of 8.25 years to minimise the correlation between linear and quadratic slope terms. Missingness was handled using full information maximum likelihood. Analyses used weights to ensure sample representativeness and allowed for the complex survey design. Indicator cut-offs applied to assess absolute model fit were <0.06 for the root mean square error of approximation and <0.08 for the standardised root mean residual.^27^

We first assessed the effects of family adversity class on symptom trajectories (using Low Risk as the reference group), adjusting for covariates (Model 1). We then assessed moderating effects of centre-based childcare, initially by adding main effects and family adversity × centre-based childcare interaction terms (Model 2). In order to show effect modifications clearly, we present the difference in outcome associated with adversity sub-type at ages 4, 6, 8, 10 and 12 years, stratified by centre-based childcare exposure.

Supplementary analyses capitalized on the availability of additional sources of outcome information from teachers and children at later time points, reducing the threat of shared variance to a causal interpretation of exposure-outcome associations.

## Results

Higher risk classes had more externalizing and internalizing symptoms than Low Risk children at each timepoint measured: table 1 shows mean symptoms, and percentages of children with severe symptoms according to adversity class. Mean scores for internalizing and externalizing symptoms were relatively low (5.6 and 2.2 for externalizing and internalizing symptoms at age 46 months), meaning that the majority of children displayed few symptoms. Between ages 46 and 152 months, there was a small decline in mean externalizing symptoms (to 4.4), and a small increase in mean internalizing symptoms (to 3.5). The prevalence of severe externalizing symptoms fell moderately from 18% at 46 months to 13% by age 152 months, whereas severe internalizing problems increased, from 6 to 19%. Mean scores were higher in the higher risk adversity classes, and there were large differences in the prevalence of severe problems: for example, compared to the Low Risk group, the prevalence of severe internalizing and externalizing problems were 4–6 times higher in Multiple Adversities.

The trajectories for externalizing and internalizing symptoms had small positive quadratic terms, indicating that the decline in externalizing symptoms (discussed above and shown in the linear term in table 2) levelled off slightly towards the end of the study period, while the increase in internalizing symptoms grew slightly faster. After covariate adjustment, higher risk classes had higher externalizing and internalizing mean scores at the midpoint of 8.25 years than the Low Risk class, with the Multiple Adversities group displaying the greatest disadvantage (table 2, stage 1). The three high-risk adversity classes also saw a steeper linear increase in internalizing scores over time than the Low Risk class, again with the Multiple Adversities group faring worst. Adding non-childcare hours and use of centre-based childcare to this model (table 2, stages 2–3) produced little change in estimated effects, suggesting that neither childcare type mediated effects of family adversity class on children’s symptoms. Centre-based childcare was associated with higher externalizing symptoms at 8.25 years, but had no association with internalizing symptoms.

**Table 2:** Adjusted associations between family adversity class and children’s externalizing and internalizing symptom trajectories

		Stage 1 adjusted baseline covariates		Stage 2 adjusted non-centre hours		Stage 3 adjusted centre use	
		Slope coeff (95% CI)	*P*-values		*P*-values	Slope coeff (95% CI)	*P*-values
Externalizing symptoms							
Intercept (8.25 years)							
Adversity class	Poor Maternal Health	1.47 (1.19 to 1.75)	<0.001	1.47 (1.19 to 1.75)	<0.001	1.47 (1.18 to 1.75)	<0.001
	Economic Hardship	1.20 (0.61 to 1.79)	<0.001	1.19 (0.59 to 1.80)	<0.001	1.23 (0.62 to 1.84)	<0.001
(ref = Low Risk)	Multiple Adversities	2.63 (1.92 to 3.33)	<0.001	2.62 (1.92 to 3.32)	<0.001	2.64 (1.95 to 3.34)	<0.001
	Non-centre hours			–0.01 (–0.12 to 0.10)	0.852	0.01 (–0.09 to 0.12)	0.792
	Centre use (yes)					0.26 (0.00 to 0.52)	0.052
Linear slope							
Adversity class	Poor Maternal Health	–0.01 (–0.05 to 0.04)	0.820	–0.01 (–0.05 to 0.03)	0.787	–0.01 (–0.05 to 0.03)	0.795
	Economic Hardship	–0.01 (–0.08 to 0.07)	0.905	–0.01 (–0.09 to 0.07)	0.737	–0.01 (–0.09 to 0.07)	0.731
(ref = Low Risk)	Multiple Adversities	0.02 (–0.07 to 0.12)	0.632	0.02 (–0.08 to 0.11)	0.750	0.02 (–0.08 to 0.11)	0.744
	Non-centre hours			–0.01 (–0.03 to 0.00)	0.045	–0.01 (–0.03 to 0.00)	0.048
	Centre use (yes)					0.00 (–0.03 to 0.03)	0.900
Quadratic slope							
Adversity class	Poor Maternal Health	–0.01 (–0.02 to 0.00)	0.103	–0.01 (–0.02 to 0.00)	0.103	–0.01 (–0.02 to 0.00)	0.102
	Economic Hardship	0.00 (–0.02 to 0.02)	0.957	0.00 (–0.03 to 0.02)	0.888	0.00 (–0.03 to 0.02)	0.909
(ref = Low Risk)	Multiple Adversities	–0.01 (–0.04 to 0.02)	0.417	–0.01 (–0.05 to 0.02)	0.388	–0.01 (–0.05 to 0.02)	0.393
	Non-centre hours			0.00 (–0.01 to 0.00)	0.512	0.00 (–0.01 to 0.00)	0.587
	Centre use (yes)					0.00 (–0.01 to 0.01)	0.473
Internalizing symptoms							
Intercept (8.25 years)							
Adversity class	Poor Maternal Health	1.25 (0.98 to 1.53)	<0.001	1.25 (0.98 to 1.52)	<0.001	1.25 (0.98 to 1.52)	<0.001
	Economic Hardship	0.94 (0.49 to 1.39)	<0.001	0.90 (0.45 to 1.35)	<0.001	0.90 (0.44 to 1.35)	<0.001
(ref = Low Risk)	Multiple adversities	2.61 (2.00 to 3.22)	<0.001	2.57 (1.95 to 3.20)	<0.001	2.57 (1.95 to 3.20)	<0.001
	Non-centre hours			–0.06 (–0.16 to 0.04)	0.246	–0.06 (–0.16 to 0.04)	0.218
	Centre use (yes)					–0.03 (–0.21 to 0.15)	0.713
Linear slope							
Adversity class	Poor Maternal Health	0.09 (0.04 to 0.15)	0.001	0.09 (0.04 to 0.14)	0.001	0.09 (0.04 to 0.14)	0.001
	Economic Hardship	0.07 (–0.01 to 0.16)	0.093	0.07 (–0.02 to 0.15)	0.119	0.07 (–0.02 to 0.15)	0.117
(ref = Low Risk)	Multiple adversities	0.19 (0.09 to 0.29)	<0.001	0.18 (0.08 to 0.28)	<0.001	0.18 (0.08 to 0.28)	<0.001
	Non-centre hours			–0.01 (–0.02 to 0.01)	0.435	–0.01 (–0.02 to 0.01)	0.404
	Centre use (yes)					0.00 (–0.03 to 0.03)	0.983
Quadratic slope							
Adversity class	Poor Maternal Health	0.00 (–0.01 to 0.02)	0.627	0.00 (–0.01 to 0.02)	0.631	0.00 (–0.01 to 0.02)	0.632
	Economic Hardship	–0.01 (–0.03 to 0.01)	0.440	–0.01 (–0.03 to 0.01)	0.422	–0.01 (–0.03 to 0.01)	0.420
(ref = Low Risk)	Multiple adversities	–0.02 (–0.05 to 0.01)	0.246	–0.02 (–0.05 to 0.01)	0.238	–0.02 (–0.05 to 0.01)	0.237
	Non-centre hours			0.00 (–0.01 to 0.00)	0.670	0.00 (–0.01 to 0.00)	0.619
	Centre use (yes)					0.00 (–0.01 to 0.01)	0.865

*Note*: Models adjusted for child sex, time in neonatal unit, low birthweight; mother’s age at birth of child, ethnicity, education, smoking in pregnancy; co-resident partner and family size. Model fit stages 1–3: RMSEA = 0.054–0.055, SRMR = 0.051–0.054.

Table 3 shows how the relationship between social adversity and socio-emotional wellbeing throughout childhood varied according to centre-based childcare use [with intercepts set at the trajectory midpoint (8.25 years)]. Differences in the externalizing scores (in the Economic Hardship class relative to the Low Risk class) widened in children not receiving Centre care (by 0.04 points per year), but with centre-based childcare, the opposite was seen, with the gap between Economic Hardship and Low Risk classes decreasing (–0.09, difference *P* = 0.035). Inequalities in internalizing scores (in Multiple Adversities relative to the Low Risk) widened in children not receiving centre-based childcare (0.12 points per year) but was greater still with centre-based childcare (0.33, difference *P* = 0.034). The detrimental effects of Multiple Adversities on externalizing problems were small with very wide confidence intervals, although they appeared to increase with time for those using Centre care and decrease for those who did not. No moderating effect of Centre care was seen on either Poor Maternal Health class trajectory. Note that there were no differences according to Centre-based childcare exposure for trajectory intercepts at 8.25 years, or for quadratic slope terms.

**Table 3:** Effect of family adversity class on externalizing and internalizing symptom trajectories, according to centre-based childcare use

		No centre-based childcare (*n* = 1708)				Centre-based childcare (*n* = 1817)				
	Effect of adversity class (ref = low risk)	Slope Coeff	95% CI			Slope Coeff	95% CI			Comparison
Externalizing symptoms			Lower	Upper	*p*-values		Lower	Upper	*P*-value	*P*-value
Intercept (8.25 years)	Poor Maternal Health	1.53	1.04	2.02	<0.001	1.49	1.10	1.88	<0.001	0.898
	Economic Hardship	1.53	0.79	2.26	<0.001	1.07	0.35	1.80	0.004	0.245
	Multiple adversities	2.28	1.31	3.25	<0.001	3.41	2.46	4.36	<0.001	0.107
Linear slope	Poor Maternal Health	0.01	–0.04	0.06	0.756	–0.02	–0.08	0.05	0.599	0.590
	Economic Hardship	**0.04**	–**0.05**	**0.14**	**0.388**	–**0.09**	–**0.18**	**0.01**	**0.082**	**0.035**
	Multiple adversities	–0.02	–0.15	0.11	0.767	0.08	–0.06	0.22	0.252	0.302
Quadratic slope	Poor Maternal Health	–0.02	–0.04	0.00	0.017	0.00	–0.02	0.02	0.978	0.067
	Economic Hardship	–0.01	–0.03	0.02	0.575	0.00	–0.03	0.04	0.837	0.576
	Multiple adversities	–0.01	–0.05	0.03	0.524	–0.02	–0.07	0.03	0.380	0.773
Internalizing symptoms										
Intercept (8.25 years)	Poor Maternal Health	1.20	0.75	1.65	<0.001	1.29	0.89	1.70	<0.001	0.806
	Economic Hardship	0.85	0.28	1.43	0.004	1.03	0.45	1.61	0.001	0.689
	Multiple adversities	2.33	1.58	3.07	<0.001	3.04	2.03	4.05	<0.001	0.285
Linear slope	Poor Maternal Health	0.11	0.04	0.17	0.001	0.07	–0.01	0.14	0.067	0.419
	Economic Hardship	0.10	0.00	0.19	0.061	0.05	–0.05	0.16	0.309	0.504
	Multiple adversities	**0.12**	–**0.01**	**0.24**	**0.076**	**0.33**	**0.17**	**0.48**	<0.001	**0.034**
Quadratic slope	Poor Maternal Health	0.00	–0.02	0.02	0.710	0.00	–0.02	0.02	0.822	0.975
	Economic Hardship	–0.01	–0.04	0.02	0.532	–0.01	–0.03	0.02	0.654	0.796
	Multiple adversities	–0.02	–0.06	0.02	0.247	–0.01	–0.05	0.04	0.792	0.470

Note: total *n* = 3525 as 36 cases with incomplete information on centre-based childcare were excluded. Intercepts were set at the trajectory midpoint (8.25 years). Models adjusted for child sex, time in neonatal unit, low birthweight; mother’s age at birth of child, ethnicity, education, smoking in pregnancy; co-resident partner and family size, constraining covariate effects to be identical in each group. Model fit RMSEA 0.051, SRMR 0.057. Figures in bold highlight statistically significant (*P* < 0.05) moderation effects.

**Figure 1:**
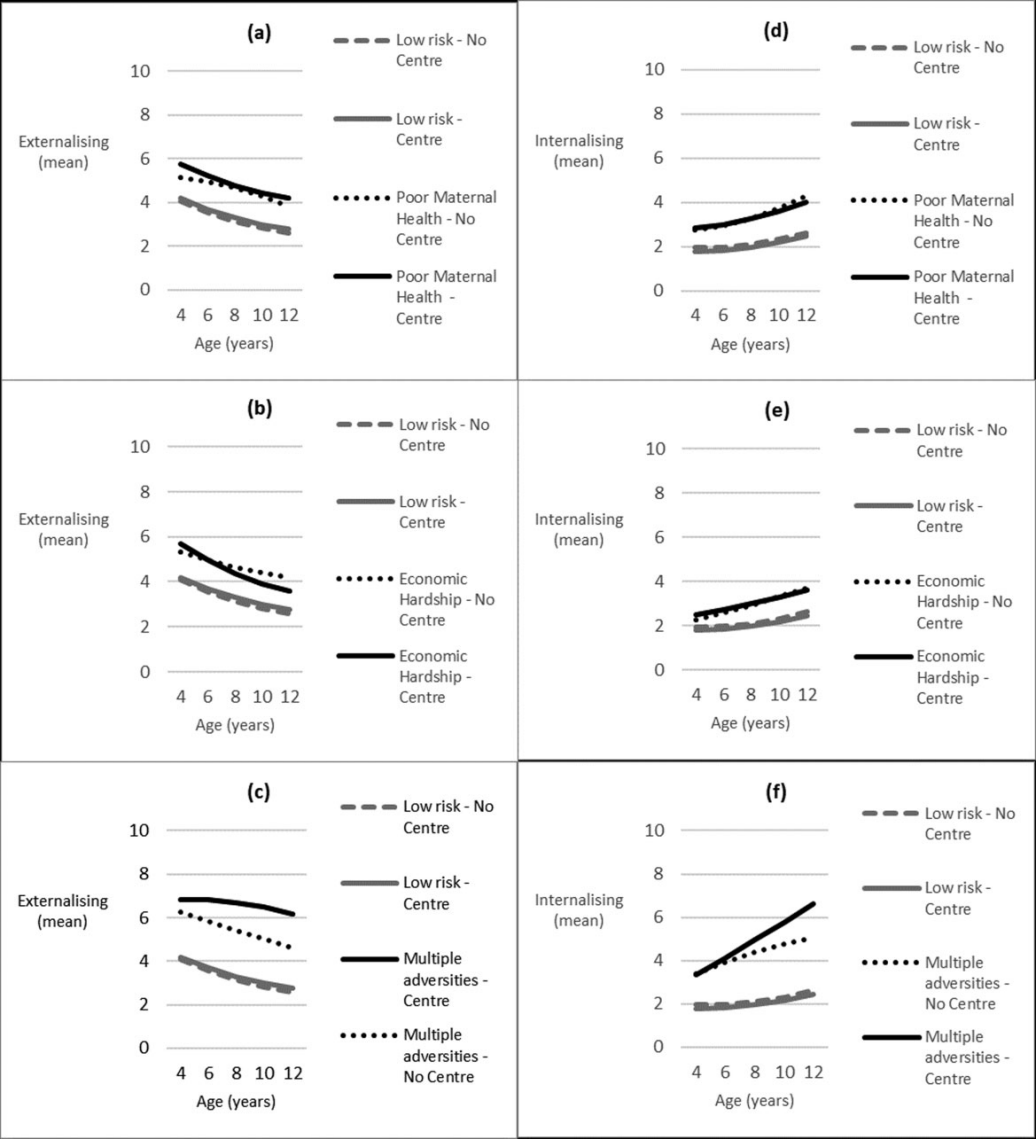
Predicted externalizing and internalizing symptoms at ages 4, 6, 8, 10 and 12 according to family adversity class and Centre care exposure in early childhood. Note: Figures show mean predicted symptoms at ages 4, 6, 8, 10 and 12, adjusted for child sex, time in neonatal unit, low birthweight; mother’s age at birth of child, ethnicity, education, smoking in pregnancy; co-resident partner and family size, constraining covariate effects to be identical in each group (no Centre care vs. Centre care)

Differences in adversity inequalities according to early childhood Centre-based childcare exposure became clearer as children grew older. Figure 1 illustrates this, after resetting trajectory intercepts to ages 4, 6, 8, 10 or 12 years. Centre-based childcare did not moderate the effects of ‘Poor Maternal Health’ on internalizing or externalizing symptoms (figure 1a, d). However, centre-based childcare appeared to alleviate inequalities by Economic Hardship in externalizing symptoms. By age 12, the difference in externalizing symptoms between Economic Hardship and Low Risk among children experiencing Centre-based childcare was 0.81, compared to 1.60 for those not receiving Centre care (*P* = 0.105, figure 1b). In contrast, centre-based childcare appeared to exacerbate the impact of Multiple Adversities on socio-emotional wellbeing. By age 12, the difference in internalizing symptoms between Multiple Adversities and Low Risk was 4.19 among children experiencing Centre care, compared to 2.43 among those not receiving Centre care (*P* = 0.014, figure 1f). For externalizing symptoms, the equivalent differences were 3.41 in those attending Centre care and 2.05 among those who were not (*P* = 0.073, figure 1c).

Supplementary analyses of symptoms at 122–152 months modelled as latent constructs, drawing on teacher and child as well as parent information, confirmed trajectory findings. Centre-based childcare reduced the effect of Economic Hardship on externalizing symptoms (interaction term coefficient –.86, *P* = 0.053) and increased the effect of Multiple Adversities on internalizing symptoms (interaction term coefficient 1.28, *P* = 0.025) and externalizing symptoms (0.84, *P* = 0.164), details on request.

## Discussion

We identified a majority low-risk group and three distinct high-risk patterns of family adversity in early childhood, drawing on data from a nationally representative population sample. In many respects, the effect of these different classes of adversity on middle childhood socio-emotional outcomes conforms to those expected from cumulative risk models. The highest risks to both externalizing and internalizing problem development were posed by Multiple Adversities, encompassing very high rates of poverty and poor maternal health, and many aspects of maladaptive family functioning. Our findings resemble those of similar studies using population samples, which found a majority group of low-risk families, and between two and four additional groups of families with different higher risk profiles, including a ‘Multiple Adversities’ group with the strongest negative impact on socio-emotional functioning.^5^^,^^6^^,^^8^ We found that the negative impacts of persistent poverty without other adversities, and of maternal health problems without extensive poverty, were similar in magnitude. This contrasts with some studies suggesting that resource-related risks alone have little impact on children’s socio-emotional development,^5^^,^^7^ but agrees with other work indicating effects of both economic and parental health domains of adversity.^8^^,^^13^^,^^28^^,^^29^

Our study goes beyond existing work on configurations of family adversity and children’s socio-emotional outcomes, by investigating moderation effects for early childhood centre-based childcare. The moderation effects found are not, however, readily explicable in terms of cumulative adversity risk. Centre-based childcare had a protective effect on externalizing symptoms among children from the Economic Hardship class, but (despite similar cumulative risk) children from families characterized by maternal health problems such as depression and stress did not experience differential benefits of centre-based childcare. Moreover, children experiencing multiple adversities including both poor maternal mental health and economic hardship appeared to be adversely affected by centre-based childcare. Maternal depression may, especially when experienced in combination with other adversities such as economic strain, substance abuse and inter-parental conflict, produce insecure attachment.^30–32^: this in turn has been linked to later behavioural and emotional symptoms.^33^^,^^34^ Childcare, especially centre-based, may aggravate insecure attachment^35^: thereby increasing, rather than reducing, inequalities in socio-emotional outcomes. Maternal depression may also affect and interact with poor family functioning to adversely influence children’s temperament,^36^ and centre-based childcare has been shown to exacerbate links between difficult temperamental characteristics and later behavioural and emotional symptoms.^37^^,^^38^ Our study was not, however, able to examine centre-based childcare quality, which may help to explain our findings if families experiencing multiple adversities accessed lower quality childcare placements.

Our findings extend existing work on differential effects of centre-based childcare, which have relatively short follow-up periods and focus on outcomes around transition to primary school.^11–16^^,^^39^ In our study, differential effects of early childhood Centre care became clearer at older ages. By age 12, centre-based childcare halved the detrimental effect of Economic Hardship on externalizing symptoms but increased the effect of Multiple Adversities on internalizing and internalizing symptoms by around 60–70%. It is not clear why the effects of Economic Hardship were only seen for externalizing and not internalizing symptoms. Low statistical power for small subgroups may have limited our ability to detect effects countering trends for all children.

Our study has several strengths and limitations, in addition to those already noted. We used data from a large representative birth cohort covering a 12-year study period with detailed annual information for the first six years of the child’s life. The use of survey weights to counteract differential attrition improves the generalizability of our findings. However, we relied on mothers for most information, and the perspectives of fathers would be a valuable contribution to future research. Supplementary analyses incorporating teacher and child information corroborated our main findings and lend support to a causal interpretation. Nonetheless, we cannot rule out the possibility that unmeasured differences between families contributed to the associations found. Many risk factors are likely to have tracked from before our 10-month study baseline: further work could examine earlier factors such as maternal pre- and post-natal depression.

Despite accumulated evidence suggesting formal childcare has long-lasting benefits for children from lower socio-economic groups,^10^ our study points to the need to consider family circumstances in more detail when assessing likely protective and harmful effects of centre-based care. Additional research is required to explore mechanisms underlying the differential effects of centre-based childcare identified here. Benefits may relate to the structured care environment,^10^ while adverse effects may stem from a poorly managed transition to Centre care, negative interactions with a large peer group and lower-quality caregiver attention.^9^^,^^40^ Further consideration should be given towards tailoring centre-based childcare provision to meet the needs of all children, regardless of family circumstances. At the same time, centre-based childcare should not be viewed as a universal solution to the problems of early childhood adversity, especially among children from multiply disadvantaged families, who are likely to benefit from renewed efforts to tackle the root causes of their adversities.

## Supplementary data

Supplementary data are available at *EURPUB* online.

## Supplementary Material

ckab006_Supplementary_Data
