# Supplementary material for: Can centre-based childcare buffer against the negative effects of family adversity on child socio-emotional wellbeing?
Source: Eur J Public Health. 2021 Feb 7;31(3):474–81. doi: 10.1093/eurpub/ckab006 (PMC7611253; doi:10.1093/eurpub/ckab006)
Supplement: ckab006_Supplementary_Data [file ckab006_supplementary_data.zip › ejph-2020-10-om-1310-File003.docx]

**Supplementary File**

**Information on additional outcomes used in supplementary analyses**

Teacher-reported information

Teachers provided information using the SDQ at 122 months.

Child-reported information

Children provided information on externalising problems using the SDQ attentional subscale at 152 months, and one item devised for the GUS study on misbehaving at school at 152 months (‘How often do you misbehave or cause trouble in class?’ with responses on a four-point scale: never, sometimes, often always). For internalising problems, children provided information on peer problems at 122 months using three items devised for the GUS study on victimisation, Cronbach’s alpha =.80, (‘How often do other children pick on you by… (a) calling you names or making fun of you, (b) leaving you out of games, (c) shoving, pushing or hitting?’ with responses on a 5-point scale: most days, about once a week, about once a month, every few months, never); and on mental wellbeing at 152 months using nine items from the Kidscreen Health-Related Quality of Life scale [1], Cronbach’s alpha =.79.

**Supplementary tables**

**Table S1 Characteristics of the Baseline, Early Childhood and Main Analysis Samples**

|  |  | Baseline sample (n=5217) |  | Early childhood sample (n=3928) |  | Analysis sample (n=3561) |
| --- | --- | --- | --- | --- | --- | --- |
|  |  | % (column) |  | % (column) |  | % (column) |
| Child gender | Male | 51.5 |  | 51.7 |  | 51.6 |
| Mother's age at birth of child (years) | <20 | 7.8 |  | 7.8 |  | 8.1 |
|  | 20-29 | 42.1 |  | 41.8 |  | 41.4 |
|  | 30-39 | 46.8 |  | 47.3 |  | 47.4 |
|  | 40+ | 3.4 |  | 3.1 |  | 3.1 |
| Mother's highest education qualification level | None/Lower level Standard Grades | 16.7 |  | 15.9 |  | 15.8 |
|  | Upper level Standard Grades | 26.7 |  | 26.8 |  | 26.9 |
|  | Highers | 30.3 |  | 31.0 |  | 31.0 |
|  | Degree | 26.3 |  | 26.3 |  | 26.3 |
| Mother's ethnic group | Minority | 4.0 |  | 3.6 |  | 3.4 |
| Co-resident partner at baseline | No | 20.3 |  | 20.5 |  | 20.7 |
| Household equivalised income quintile | 1 (lowest) | 21.5 |  | 21.6 |  | 21.4 |
|  | 2 | 20.8 |  | 20.3 |  | 20.5 |
|  | 3 | 18.1 |  | 18.6 |  | 18.5 |
|  | 4 | 21.0 |  | 20.7 |  | 21.1 |
|  | 5 (highest) | 18.5 |  | 18.8 |  | 18.5 |
|  |  |  |  |  |  |  |
|  |  |  |  |  |  |  |
|  |  |  |  |  |  |  |
| Note: Sweep 1 (age 10 months) survey weights were applied to percentages in the Baseline sample, sweep 3 (age 34 months) weights to percentages in the Early Childhood Sample, and modified sweep3 weights (=sweep 3 x inverse probability of inclusion) to percentages in the Main Analysis sample | | | | | | |

**Table S2 Indicators of Family Adversity: information on measurement, overall prevalence and predicted probability in family adversity subtypes**

|  |  |  |  |  | **Prevalence** | Predicted probability of indicator in each family adversity class | | | |
| --- | --- | --- | --- | --- | --- | --- | --- | --- | --- |
| **Indicator group** | **Indicator** | **Time points** | **Information** | **Recode to create binary item** | **Total sample (%)** | Multiple | Health | Poverty/discord | Low risk |
| Economic | Low income | 10, 22, 34 months | Equivalised net household income | Income <60% of median at 2 or 3 timepoints | 24.8 | 0.93 | 0.22 | 0.88 | 0.10 |
|  | Means-tested benefits | 10, 22, 34 months | Receipt of income support | At 2 or 3 timepoints | 14.0 | 0.92 | 0.04 | 0.85 | 0.00 |
|  | Workless household | 10, 22, 34 months | Mother and (if applicable) partner unemployed | At 2 or 3 timepoints | 11.8 | 0.85 | 0.00 | 0.66 | 0.00 |
|  | Financial difficulties | 10, 22, 34 months | “Which of the phrases on this card comes closest to your feelings about your household income these days?”: responses 1 Living very comfortably on present income 2 Living comfortably on present income 3 Coping on present income 4 Finding it difficult on present income 5 Finding it very difficult on present income. | Difficulties (responses 4 or 5) at 2 or 3 timepoints | 11.9 | 0.51 | 0.21 | 0.21 | 0.04 |
|  |  |  |  |  |  |  |  |  |  |
| Family relationships | Interparental conflict | 22 months | For mothers with a co-residing partner, this used 3 items, standardised Cronbach’s alpha= .68: (a) "How often do you and your partner argue?" Responses (1) More than once a week, (2) Once a week or less, (3) Not at all; (b) "How often is there anger or hostility between you and your partner?" Responses as (a); (c) "How often do you and your partner/husband disagree over issues relating to bringing up (child’s name)?" Responses (reverse-scored) (1) Never (2) Less than once a week (3) Once a week (4) Several times a week (5) Once a day (6) More than once a day. For mothers without a co-residing partner, conflict was based on mother's description of their relationship with the non-resident father: (1) Very good (2) Fairly good (3) Neither good nor bad (4) Fairly bad (5) Very bad. | For co-residing couples, conflict was coded as a score of more than one standard deviation below the mean. For mothers without a partner, conflict was based on responses 4 and 5 for the item on relationship with non-resident father. | 12.5 | 0.60 | 0.27 | 0.54 | 0.09 |
|  | Partner separation | 22, 34 months | Used information on (a) changes to household composition between sweeps of data collection, (b) partner not full-time resident reported in the list of household events that had occurred since the past year, (c) partner death reported in household events. | Loss at either timepoint | 6.5 | 0.20 | 0.09 | 0.20 | 0.02 |
|  | New partner | 22, 34 months | Based on (a) a co-resident partner being recorded, where the mother had reported no partner at the previous sweep of data collection; and/or (b) a new partner being reported in the list of household events. | New partner at either timepoint | 5.3 | 0.14 | 0.07 | 0.23 | 0.01 |
|  | Harsh parenting | 22 months | Two items: parental smacking of cohort child, or other child in household, during past year | Smacking of any child in household | 29.0 | 0.30 | 0.40 | 0.23 | 0.27 |
|  |  |  |  |  |  |  |  |  |  |
| Maternal Health | Low mental health | 10, 34 months | Mental health subscale of the short form Sf12 scale [2] | Health <1 SD below mean at either time point | 22.7 | 0.58 | 0.37 | 0.12 | 0.05 |
|  | Depression and stress | 22 months | Items on depression and stress from the Depression Anxiety Stress scale [3] | Depression/stress >1 SD above mean | 11.3 | 0.50 | 0.33 | 0.06 | 0.02 |
|  | Alcohol use | 10, 34 months | "How often, on average, do you have an alcoholic drink?" Responses scored: 1 Every day, 2 4-6 times a week, 3 2 to 3 times a week, 4 Once a week, 5 2 to 3 times a month, 6 Once a month or less, 7 Not in the last year, 8 Do not drink at all. | Alcohol consumed 4+ days/week at either time point | 8.9 | 0.03 | 0.09 | 0.01 | 0.10 |
|  | Illegal drug use | 10, 34 months | Use of one or more of the following substances in the past year: Cannabis, Amphetamines, Cocaine, Crack, Ecstasy, Heroin, Methadone, LSD, Another illegal drug. | Illegal drug use in past year at either time point | 5.8 | 0.23 | 0.07 | 0.14 | 0.03 |
|  | Low physical health | 10, 34 months | Physical health subscale of the short form Sf12 scale [2] | Health <1 SD below mean at either time point | 19.8 | 0.64 | 0.46 | 0.11 | 0.09 |
|  | Limiting illness/disability | 10, 22, 34 months | 2 items : (a) "Do you have any health problems or disabilities that have lasted or are expected to last more than a year?" , and if “yes”, (b) "Does this health problem or disability limit your ability to carry out normal day-to-day activities?" | Limiting illness/disability at any time point | 10.7 | 0.58 | 0.36 | 0.00 | 0.00 |
| Household events | Family illness/accident | 22, 34 months | Parent and/or sibling illness/accident. | Illness/accident at either time point | 5.6 | 0.13 | 0.11 | 0.04 | 0.04 |
|  | New child | 22, 34 months | New baby or older child joining household - from household events list | new baby or child in household at either time point | 22.2 | 0.21 | 0.21 | 0.19 | 0.23 |
|  | Loss of child | 22, 34 months | Child left household, or died - from household events list | child left household or died at either time point | 1.5 | 0.04 | 0.01 | 0.03 | 0.01 |
|  | Death of grandparent/ other relative | 22, 34 months | Death - in household events list | death at either time point | 15.1 | 0.17 | 0.18 | 0.17 | 0.14 |
|  | Moved house | 22, 34 months | Address change | moved at either time point | 25.5 | 0.31 | 0.29 | 0.34 | 0.23 |

Table S3 Model fit statistics for Latent Class Analyses of early childhood family adversity indicators

| Number of classes | Log likelihood | AIC | BIC | Entropy | Smallest class (%) | LMR *p*-value |
| --- | --- | --- | --- | --- | --- | --- |
| 1 | -27975.6 | 55989.2 | 56108.5 | N/A | 100 | N/A |
| 2 | -25607.6 | 51293.2 | 51537.9 | 0.93 | 19 | <.001 |
| 3 | -25209.5 | 50537.0 | 50907.2 | 0.79 | 16 | <.001 |
| **4** | **-25028.3** | **50214.5** | **50710.3** | **0.81** | **6** | **0.006** |
| 5 | -24925.5 | 50049.0 | 50670.3 | 0.84 | 6 | 0.278 |

Note: Smaller Akaike Information Criteria (AIC) and Bayesian Information Criteria (BIC) values are preferable, while Entropy values should be close to 1. The Lo, Mendell and Rubin Likelihood Ratio Test (LMR) test indicated whether a model has a better fit than the model with one fewer class

**Table S4 Associations between family adversity class and use of centre childcare**

|  |  | **Family adversity class** | | | | **Total** |
| --- | --- | --- | --- | --- | --- | --- |
| **Centre care at 10, 22 and 34 months** |  | **Multiple** | **Maternal health problems** | **Poverty** | **Low risk** |  |
| None | % | 58.2 | 48.2 | 60.0 | 47.5 | 50.0 |
|  | *n* | *102* | *279* | *193* | *1134* | *1708* |
|  |  |  |  |  |  |  |
| Low hours (average <12 hours per week) | % | 26.8 | 27.3 | 25.3 | 25.8 | 26.0 |
|  | *n* | *45* | *161* | *79* | *639* | *924* |
|  |  |  |  |  |  |  |
| High (average 12+ hours per week) | % | 15.0 | 24.5 | 14.8 | 26.7 | 23.9 |
|  | *n* | *26* | *142* | *45* | *680* | *893* |
|  |  |  |  |  |  |  |
| Total | % | 100.0 | 100.0 | 100.0 | 100.0 | 100.0 |
|  | *n* | *173* | *582* | *317* | *2453* | *3525* |

References

1. Ravens-Sieberer, U., et al., *Reliability, construct and criterion validity of the KIDSCREEN-10 score: a short measure for children and adolescents' well-being and health-related quality of life.* Qual Life Res, 2010. **19**(10): p. 1487-500.

2. Jenkinson, C. and R. Layte, *Development and testing of the UK SF-12 (short form health survey).* Journal of Health Services Research & Policy, 1997. **2**(1): p. 14-8.

3. Crawford, J.R. and J.D. Henry, *The Depression Anxiety Stress Scales (DASS): normative data and latent structure in a large non-clinical sample.* Br J Clin Psychol, 2003. **42**(Pt 2): p. 111-31.
