# Supplementary material for: Can centre-based childcare buffer against the negative effects of family adversity on child socio-emotional wellbeing?
Source: Eur J Public Health. 2021 Feb 7;31(3):474–81. doi: 10.1093/eurpub/ckab006 (PMC7611253; doi:10.1093/eurpub/ckab006)

Figure S1 Family Adversity classes in early childhood showing distribution of indicators within each class

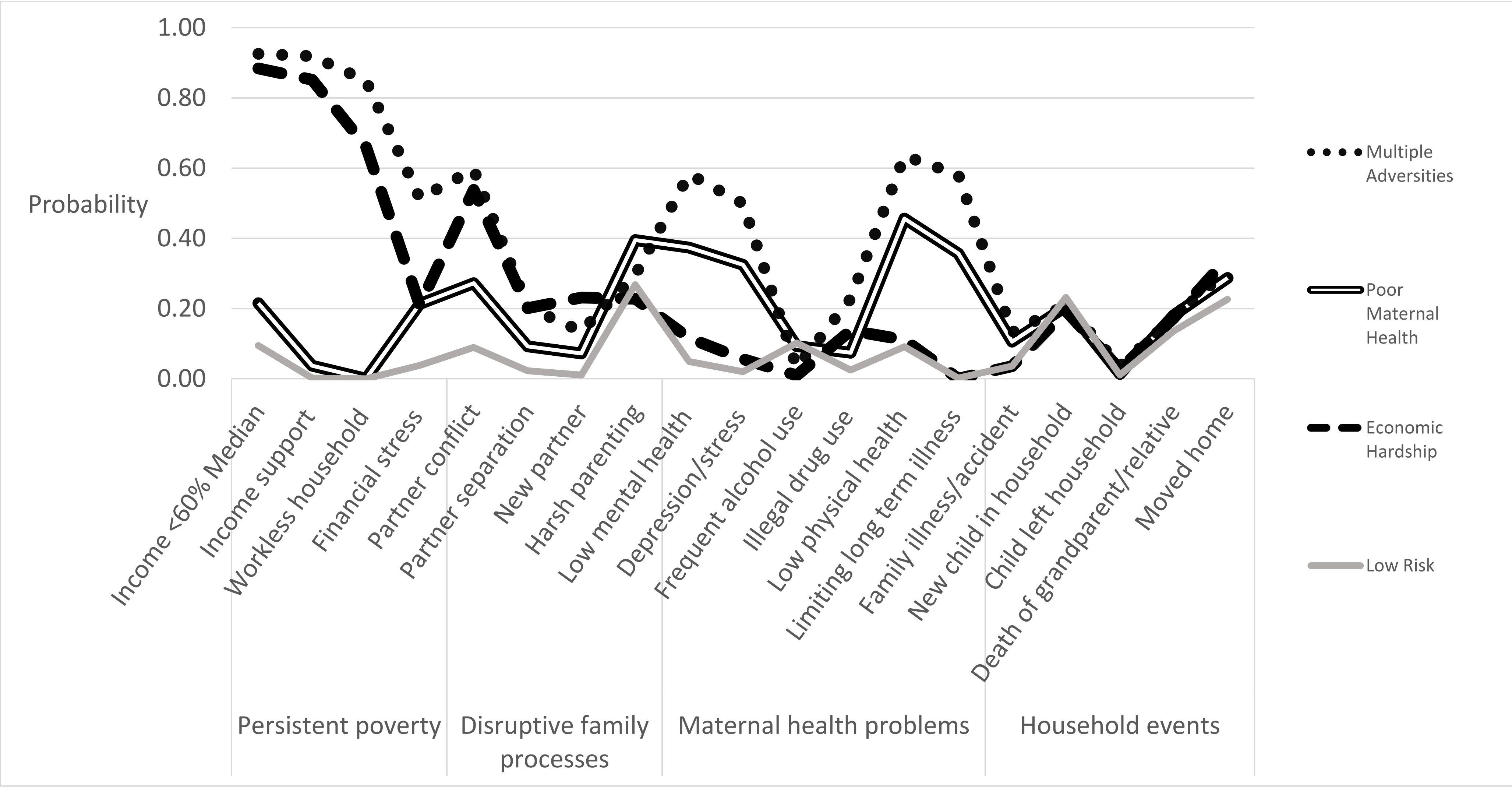

Supplement: ckab006_Supplementary_Data [file ckab006_supplementary_data.zip › ejph-2020-10-om-1310-File004.pdf]
