# Supplementary material for: Can centre-based childcare buffer against the negative effects of family adversity on child socio-emotional wellbeing?
Source: Eur J Public Health. 2021 Feb 7;31(3):474–81. doi: 10.1093/eurpub/ckab006 (PMC7611253; doi:10.1093/eurpub/ckab006)
Supplement: ckab006_Supplementary_Data [file ckab006_supplementary_data.zip › ejph-2020-10-om-1310-File005.docx]

Figure S2: Flow diagram to show how final analytic sample was arrived at

**Baseline Sample,** *n*=5217

**Early Childhood Sample** *n*=3928

**Analysis Sample** *n*=3561

(all other missing information imputed)

Excluded:

Not present at third interview (when exposure measured), *n*=1097

Multiple births, *n*=78

Respondent not the mother at all relevant sweeps *n*=114

Excluded:

No externalising and internalising information collected at any time point, *n*=367
